# Multiaxial Lenticular Stress-Strain Relationship of Native Myocardium is Preserved by Infarct-Induced Natural Heart Regeneration in Neonatal Mice

**DOI:** 10.1038/s41598-020-63324-w

**Published:** 2020-04-30

**Authors:** Hanjay Wang, Ross Bennett-Kennett, Michael J. Paulsen, Camille E. Hironaka, Akshara D. Thakore, Justin M. Farry, Anahita Eskandari, Haley J. Lucian, Hye Sook Shin, Matthew A. Wu, Annabel M. Imbrie-Moore, Amanda N. Steele, Lyndsay M. Stapleton, Yuanjia Zhu, Reinhold H. Dauskardt, Y. Joseph Woo

**Affiliations:** 1 Department of Cardiothoracic Surgery, Stanford University, Stanford, CA USA; 2 Stanford Cardiovascular Institute, Stanford University, Stanford, CA USA; 3 Department of Materials Science and Engineering, Stanford University, Stanford, CA USA; 4 Department of Mechanical Engineering, Stanford University, Stanford, CA USA; 5 Department of Bioengineering, Stanford University, Stanford, CA USA

**Keywords:** Cardiac regeneration, Myocardial infarction, Mechanical properties

## Abstract

Neonatal mice exhibit natural heart regeneration after myocardial infarction (MI) on postnatal day 1 (P1), but this ability is lost by postnatal day 7 (P7). Cardiac biomechanics intricately affect long-term heart function, but whether regenerated cardiac muscle is biomechanically similar to native myocardium remains unknown. We hypothesized that neonatal heart regeneration preserves native left ventricular (LV) biomechanical properties after MI. C57BL/6J mice underwent sham surgery or left anterior descending coronary artery ligation at age P1 or P7. Echocardiography performed 4 weeks post-MI showed that P1 MI and sham mice (n = 22, each) had similar LV wall thickness, diameter, and ejection fraction (59.6% vs 60.7%, p = 0.6514). Compared to P7 shams (n = 20), P7 MI mice (n = 20) had significant LV wall thinning, chamber enlargement, and depressed ejection fraction (32.6% vs 61.8%, p < 0.0001). Afterward, the LV was explanted and pressurized *ex vivo*, and the multiaxial lenticular stress-strain relationship was tracked. While LV tissue modulus for P1 MI and sham mice were similar (341.9 kPa vs 363.4 kPa, p = 0.6140), the modulus for P7 MI mice was significantly greater than that for P7 shams (691.6 kPa vs 429.2 kPa, p = 0.0194). We conclude that, in neonatal mice, regenerated LV muscle has similar biomechanical properties as native LV myocardium.

## Introduction

The morbidity and mortality associated with ischemic heart disease remains staggeringly high, impacting the lives of over 150 million patients worldwide^1^. Despite continued innovations in pharmacologic therapy and coronary revascularization strategies^2,3^, many adult patients who are optimally treated for myocardial infarction (MI) nevertheless develop heart failure due to ischemic cardiomyopathy^4^. During the weeks immediately following MI in adult mammals, including humans, the left ventricle (LV) wall thins due to cardiomyocyte cell death, and the nonviable muscle is gradually replaced with stiff collagen scar^5,6^. Infarct stiffening supports LV systolic function by limiting the degree of infarct bulging, but also reduces LV diastolic filling^7–9^. To compensate for reduced cardiac output, LV dilatation ensues, resulting in increased LV wall stress by Laplace’s Law, which then initiates a vicious cycle of further LV enlargement and adverse remodeling, ultimately leading to heart failure^10^. These biomechanical principles of MI evolution are well-described and are critically important to understanding the long-term function and durability of the injured ventricular muscle^9^.

Cardiac regeneration represents a promising potential solution for the treatment or prevention of heart failure after MI^11^. While adult mammals exhibit limited cardiomyocyte proliferation at baseline and after MI^12,13^, adult zebrafish impressively are capable of natural heart regeneration after myocardial injury^14^. Recent studies by our team and others have also demonstrated that newborn mice are capable of robust natural heart regeneration after MI^15–18^. This intrinsic neocardiomyogenic process, which is active in mice on postnatal day 1 (P1) but lost by postnatal day 7 (P7), results in preservation of normal LV size and function, as well as minimal replacement of LV muscle by collagen scar after MI. A similar, transient regenerative phenomenon in response to MI has also been reported in newborn rats^19^, newborn piglets^20,21^, and possibly in human neonates as well^22^.

The biomechanical properties of naturally regenerated LV muscle after MI have not been thoroughly examined. Because MI injury significantly disrupts global tissue architecture^15^, confirmation is needed that the regenerated cardiac muscle is mechanically integrated such that tissue-level biomechanical properties are optimally maintained^23^. A previous study employed a cryoinjury model in adult zebrafish, and demonstrated using micropipette aspiration that while the injured myocardium stiffened acutely after sustaining damage, myocardial biomechanics fully normalized by 5 weeks after injury in association with complete natural cardiac regeneration^24^. Thus far, however, biomechanical analyses of infarct-induced, naturally-regenerated LV tissue have not been performed using the more clinically-relevant coronary artery ligation technique in a more translationally-relevant mammalian model.

Here, we apply lenticular hydrostatic deformation analysis to study the biomechanical properties of neonatal mouse hearts after MI-induced natural cardiac regeneration. We hypothesized that, after P1 MI, regenerated mouse hearts exhibit a stress-strain relationship and composite multiaxial modulus similar to that of native, healthy LV tissue. We additionally hypothesized that, after P7 MI, mouse hearts that fail to regenerate exhibit significant tissue stiffening due to collagen scar formation.

## Results

### Confirmation of acute myocardial infarction

**Figure 1:**
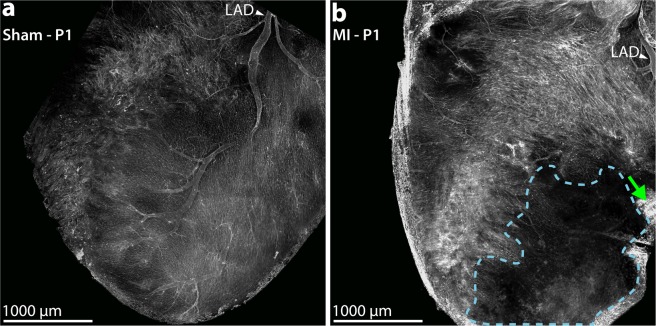
Confirmation of Neonatal Myocardial Infarction. Whole-mount confocal microscopy of fluorescent lectin-perfused neonatal mouse hearts at 1 hour after sham or myocardial infarction (MI) surgery on postnatal day 1 (P1). **(a)** For sham hearts (n = 4), fluorescent lectin signal is observed throughout the entire heart surface. The left anterior descending (LAD) coronary artery is labeled. **(b)** For MI hearts (n = 5), the LAD territory distal to the ligation suture (green arrow) is devoid of lectin signal (dotted blue trace), confirming successful LAD ligation.

Neonatal mice underwent permanent left anterior descending (LAD) coronary artery ligation or sham surgery, either at age P1 (n = 63) or age P7 (n = 46). A subset of P1 mice in the sham (n = 4) and MI groups (n = 5) were sacrificed 1 hour after surgery to confirm successful LAD ligation. Whole-mount confocal microscopy of fluorescent lectin-perfused sham hearts revealed bright signal throughout the anterior surface of the LV (Fig. 1a). All MI hearts examined, however, exhibited absence of fluorescent signal in a well-defined area distal to the site of the ligation suture, confirming successful LAD ligation and acute MI (Fig. 1b).

### Confirmation of age-dependent cardiac regeneration

**Figure 2:**
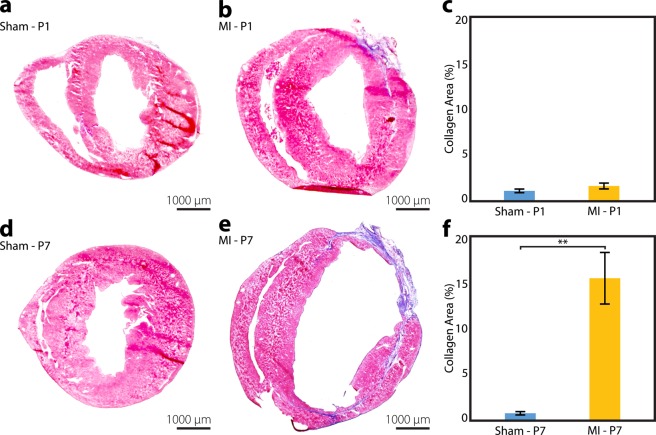
Infarct Size Analysis. Masson’s trichrome analysis of collagen scar formation at 4 weeks after sham or myocardial infarction (MI) surgery, performed in neonatal mice on postnatal day 1 (P1) or postnatal day 7 (P7). **(a,b)** Minimal intramural collagen scar was detected in the P1 sham (n = 4) and P1 MI hearts (n = 6). **(c)** No difference was observed among P1 sham and P1 MI hearts in the quantified percentage of collagen area relative to total left ventricular area. **(d,e)** Transmural collagen scar and adverse left ventricular remodeling were observed in the P7 MI hearts (n = 3) compared to sham controls (n = 3). **(f)** Percent collagen area relative to total left ventricular area was significantly greater for P7 MI hearts compared to P7 sham hearts. Data presented as mean ± standard error and compared using two-sample t-test. **Indicates p < 0.01.

Explanted hearts from mice assigned for histological analyses were examined at 4 weeks after P1 or P7 surgery using Masson’s trichrome staining or 5-ethynyl-2′-deoxyuridine (EdU) labeling with troponin immunohistochemistry. Masson’s trichrome stain revealed minimal intramyocardial collagen scar formation in P1 MI hearts (n = 6) compared to P1 sham hearts (n = 4), suggesting cardiac regeneration after P1 MI (Fig. 2a,b). Quantified collagen area as a percentage of total LV area was similar between the P1 MI and P1 sham hearts (1.8 ± 0.3% vs 1.3 ± 0.2%, respectively, p = 0.2415, Fig. 2c). In contrast, P7 MI hearts (n = 3) exhibited severe adverse LV remodeling and significant deposition of collagen within the thinned anterolateral LV wall compared to P7 sham hearts (n = 3), indicating absence of cardiac regeneration after P7 MI (Fig. 2d,e). Percent collagen area was significantly greater for P7 MI hearts than P7 sham hearts (15.6 ± 2.8% vs 0.9 ± 0.2%, respectively, p = 0.0064, Fig. 2f).

**Figure 3:**
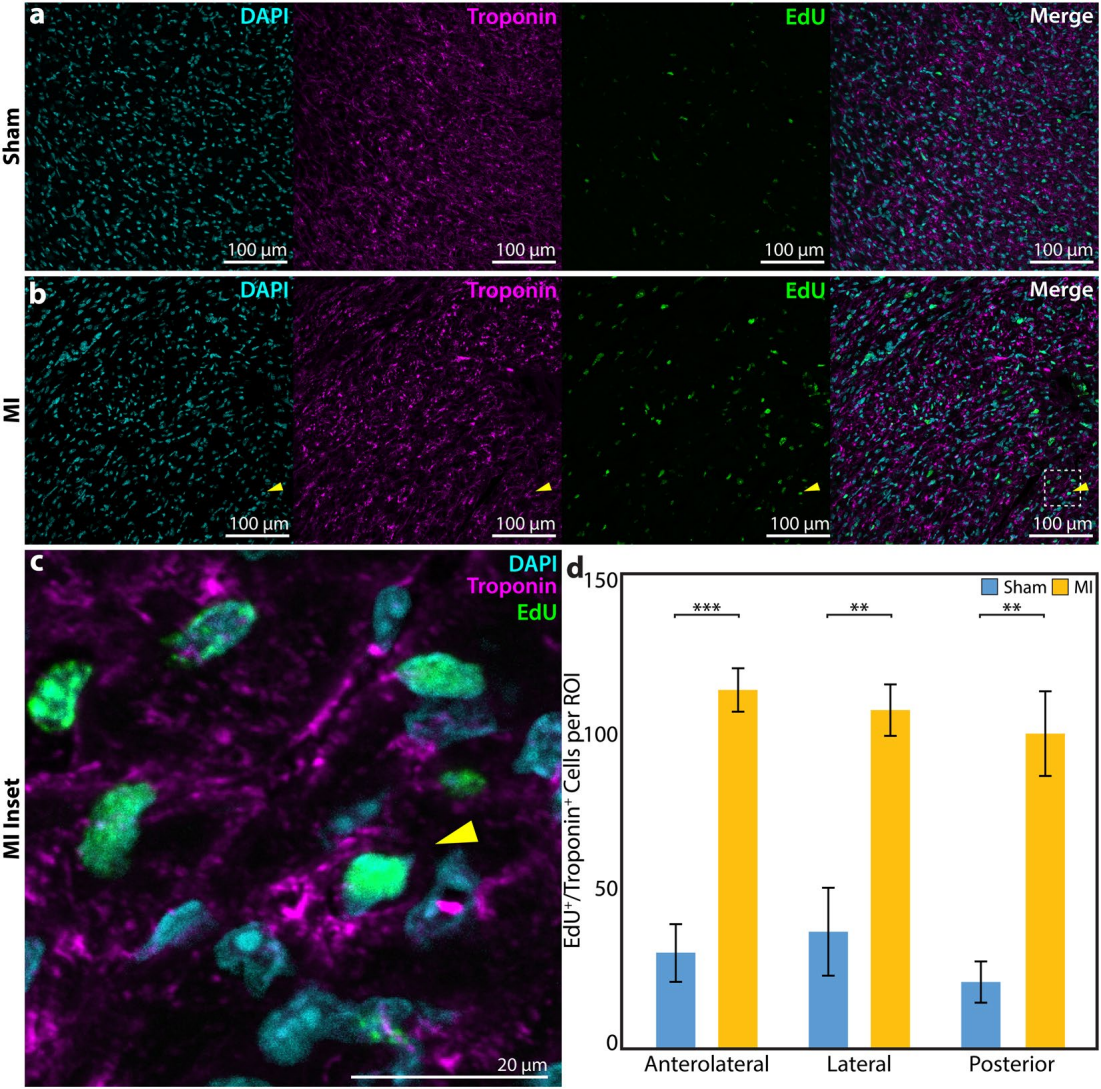
Assessment of Cardiomyocyte Proliferation. 5-ethynyl-2′-deoxyuridine (EdU) signal after sham or myocardial infarction (MI) surgery in neonatal mice on postnatal day 1. **(a,b)** Within the anterolateral left ventricular (LV) wall, compared to sham hearts, the MI hearts appeared to exhibit a greater density of EdU signal in the DAPI-stained nuclei of troponin^+^ cardiomyocytes, with **(c)** inset (dotted box) showing an EdU^+^/troponin^+^ cardiomyocyte (yellow arrow). **(d)** EdU^+^/troponin^+^ cells were significantly more prevalent per 20X region of interest (ROI) in MI hearts (n = 5) compared to sham hearts (n = 4) in all cardiac regions examined. Data presented as mean ± standard error and compared using two-sample t-test. **Indicates p < 0.01. ***Indicates p < 0.001.

To verify that natural cardiac regeneration occurs after P1 MI, a subset of P1 MI mice (n = 5) and P1 sham mice (n = 4) assigned for histological analysis received EdU, a thymidine analog and marker of DNA replication during cell proliferation, at 1, 7, and 14 days after surgery. At 4 weeks after surgery, EdU signal was assessed for both P1 MI and P1 sham hearts in three regions of the LV: (1) the anterolateral LV wall, representing the potential infarct zone after MI; (2) the lateral LV wall, representing the potential border zone after MI; and (3) the posterior LV wall, representing uninjured LV tissue after MI. EdU was found to be incorporated into the DAPI-stained nuclei of troponin^+^ cardiomyocytes at a significantly greater density in the anterolateral LV wall of P1 MI hearts compared to P1 sham hearts (Fig. 3a–c). The number of EdU^+^/troponin^+^ cardiomyocytes in each 20X region of interest was quantified for P1 MI and P1 sham hearts, respectively, in the anterolateral LV wall (114.5 ± 6.9 cells/field vs 30.8 ± 9.1 cells/field, p = 0.0001), as well as in the lateral LV wall (108.1 ± 8.2 cells/field vs 37.5 ± 14.0 cells/field, p = 0.0025), and the posterior LV wall (100.6 ± 13.5 cells/field vs 21.4 ± 6.6 cells/field, p = 0.0019), suggesting a state of global LV cardiomyocyte proliferation after P1 MI (Fig. 3d).

### Neonatal heart regeneration preserves cardiac geometry and function after MI

All P1 and P7 mice assigned for biomechanical testing underwent echocardiography at 4 weeks after surgery (Table 1). Among P1 MI (n = 22) and P1 sham mice (n = 22), no significant differences were observed in LV wall thickness in diastole (LVWTd 0.61 mm vs 0.64 mm, p = 0.4700), LV internal diameter in systole (LVIDs 2.32 mm vs 2.28 mm, p = 0.6659) or diastole (LVIDd 3.36 mm vs 3.34 mm, p = 0.8693), or LV ejection fraction (59.6% vs 60.7%, p = 0.6514), respectively, suggesting robust cardiac regeneration after P1 MI. Compared to P7 sham mice (n = 20), P7 MI mice (n = 20) exhibited hearts which were significantly thinned (LVWTd 0.40 mm vs 0.71 mm, p < 0.0001) and dilated (LVIDs 3.69 mm vs 2.27 mm, p < 0.0001; LVIDd 4.34 mm vs 3.35 mm, p < 0.0001), with dramatically reduced LV ejection fraction (32.6% vs 61.8%, p < 0.0001), indicating absence of cardiac regeneration after P7 MI.

**Table 1:** Echocardiography.

P1 Mice	P1 Sham (n = 22)	P1 MI (n = 22)	P-value
Heart Rate (bpm)	453.4 ± 14.7	435.0 ± 15.8	0.3982
LVWTd (mm)	0.64 ± 0.02	0.61 ± 0.03	0.4700
LVIDs (mm)	2.28 ± 0.06	2.32 ± 0.07	0.6659
LVIDd (mm)	3.34 ± 0.07	3.36 ± 0.07	0.8693
Ejection Fraction (%)	60.7 ± 1.6	59.6 ± 1.7	0.6514
P7 Mice	P7 Sham (n = 20)	P7 MI (n = 20)	P-value
Heart Rate (bpm)	433.8 ± 14.2	444.1 ± 15.2	0.6231
LVWTd (mm)	0.71 ± 0.02	0.40 ± 0.02	<0.0001
LVIDs (mm)	2.27 ± 0.09	3.69 ± 0.17	<0.0001
LVIDd (mm)	3.35 ± 0.07	4.34 ± 0.14	<0.0001
Ejection Fraction (%)	61.8 ± 2.1	32.6 ± 3.2	<0.0001

Echocardiographic assessment of cardiac geometry and function at 4 weeks after surgery. LVIDd, left ventricle internal diameter in diastole; LVIDs, left ventricle internal diameter in systole; LVWTd, left ventricle wall thickness in diastole; MI, myocardial infarction; P1, postnatal day 1; P7, postnatal day 7. Data presented as mean ± standard error and compared using two-sample t-test.

### Neonatal heart regeneration preserves native myocardial biomechanics after MI

**Figure 4:**
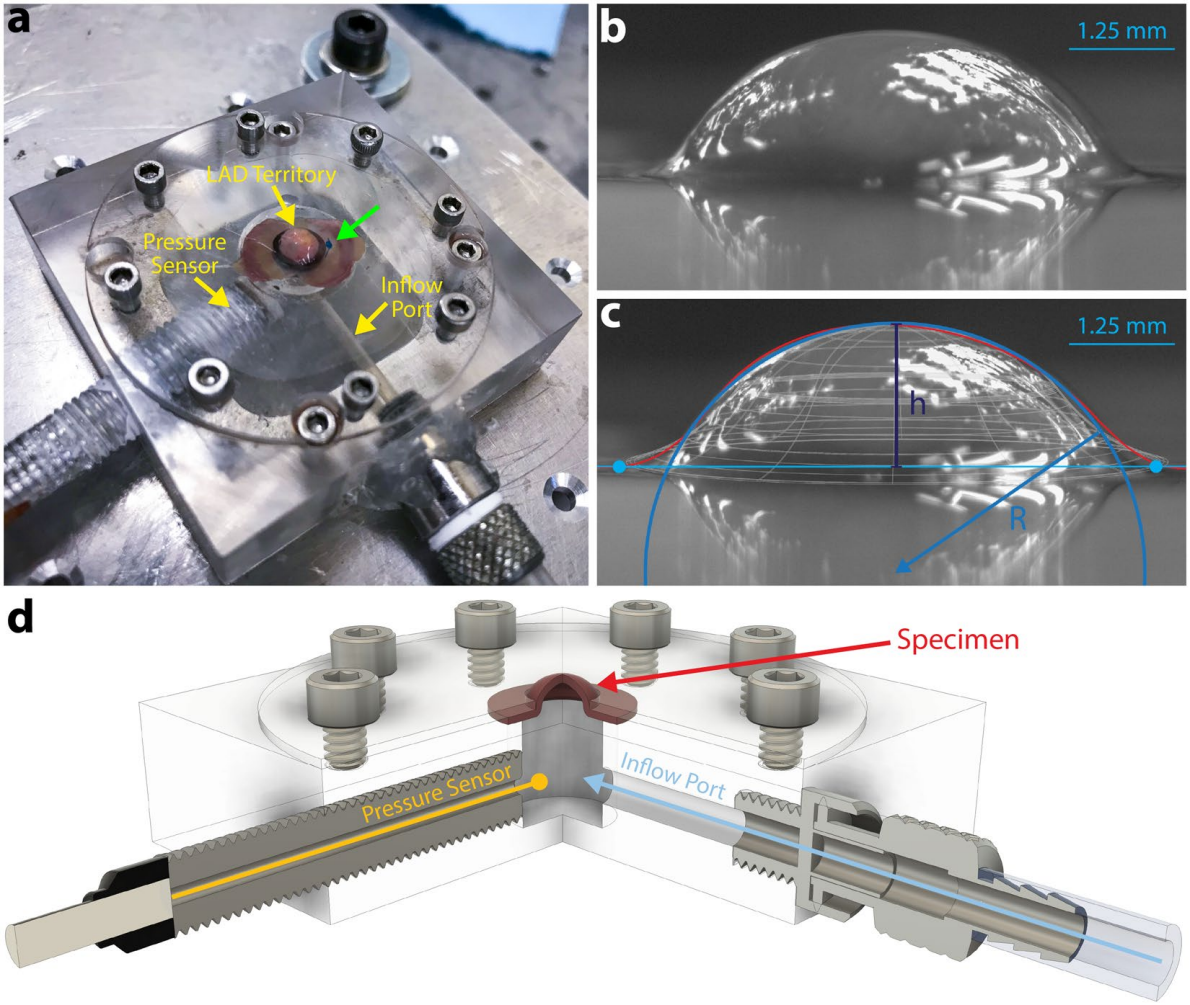
Lenticular Hydrostatic Deformation Testing. (**a**) Biomechanical testing configuration with mouse left ventricle (LV) wall sample mounted over a pressurizable, sealed chamber. The heart is mounted such that the LV territory supplied by the left anterior descending (LAD) coronary artery is centered over the orifice of the gasket (LAD ligation stitch, green arrow). The inflow port and pressure sensor are also labeled. **(b)** The LV is pressurized up to 150 mmHg, inducing lenticular deformation. **(c)** Tissue deformation (magenta trace) is modeled as the cap section of a sphere with radius, *R* (dark blue trace). The height, *h*, of the spherical cap is labeled. **(d)** Schematic illustration of lenticular hydrostatic deformation testing, created using Autodesk Fusion 360 software (https://www.autodesk.com/products/fusion-360/overview).

**Figure 5:**
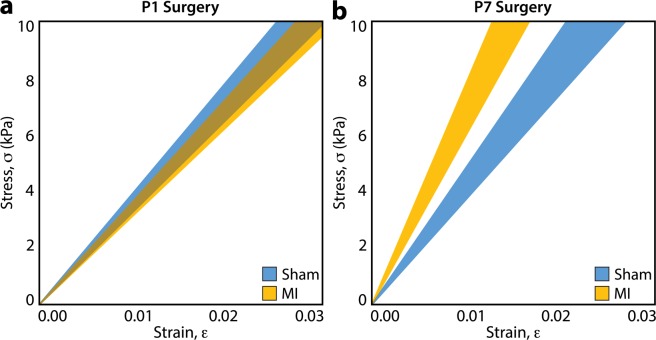
Average Stress-Strain Curves. Average stress-strain relationship of left ventricular tissue from mice after sham or myocardial infarction (MI) surgery on postnatal day 1 (P1) or postnatal day 7 (P7), derived from lenticular hydrostatic deformation testing. **(a)** After P1 surgery, sham (n = 22) and MI hearts (n = 22) exhibited similar stress-strain curves. **(b)** After P7 surgery, MI hearts (n = 20) exhibited significantly stiffer behavior than sham hearts (n = 20). Shaded region represents standard error.

At 4 weeks post-MI, biomechanical assessment of the explanted LV tissue was performed using lenticular hydrostatic deformation analysis (Fig. 4). Representative stress-strain experimental data plots derived from lenticular hydrostatic deformation testing are shown in Supplemental Fig. 1. A strong linear stress-strain relationship was observed for all sham and MI hearts in both the P1 and P7 age groups (r^2^ > 0.99 for all samples). Average stress-strain curves for the P1 sham (n = 22), P1 MI (n = 22), P7 sham (n = 20), and P7 MI hearts (n = 20) were calculated, revealing close overlap of the average P1 sham and P1 MI curves (Fig. 5a), suggestive of preserved myocardial tissue compliance after P1 MI. The average P7 MI curve, however, deviated substantially from the average P7 sham curve, indicating an increase in composite tissue stiffness after P7 MI (Fig. 5b).

**Figure 6:**
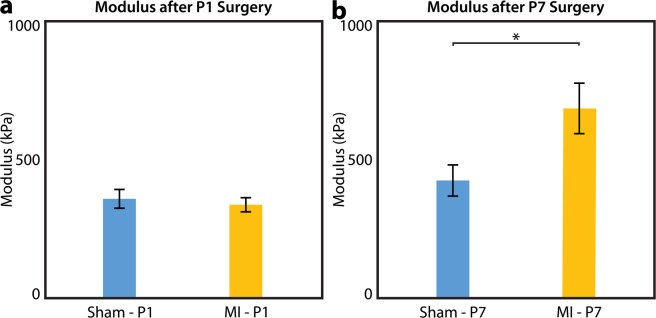
Multiaxial Modulus. Composite multiaxial modulus of left ventricular tissue from mice after sham or myocardial infarction (MI) surgery on postnatal day 1 (P1) or postnatal day 7 (P7), derived from lenticular hydrostatic deformation testing. **(a)** After P1 surgery, sham (n = 22) and MI hearts (n = 22) exhibited similar myocardial tissue compliance. **(b)** After P7 surgery, MI hearts (n = 20) exhibited significantly stiffer tissue than sham hearts (n = 20). Data presented as mean ± standard error and compared using two-sample t-test. *Indicates p < 0.05.

A composite multiaxial tissue modulus was calculated for all samples tested. P1 MI and P1 sham hearts exhibited similar LV tissue compliance (341.9 ± 25.5 kPa vs 363.4 ± 33.9 kPa, p = 0.6140, Fig. 6a), while P7 MI hearts were significantly stiffer than P7 sham hearts (691.6 ± 91.5 kPa vs 429.2 ± 56.4 kPa, p = 0.0194, Fig. 6b), thus confirming preservation of myocardial biomechanics after P1 MI but not after P7 MI.

## Discussion

Our study is the first to investigate LV biomechanics after MI induced by coronary artery ligation in a neonatal mammalian model of natural heart regeneration. Using lenticular hydrostatic deformation analysis, we observed that the endogenous neocardiomyogenesis program that acts in response to MI in P1 mice fully preserves native LV biomechanical properties at 4 weeks after injury. After P7 MI, however, cardiac regeneration does not occur and the LV region at risk stiffens significantly in association with replacement of cardiac muscle by collagen scar, similar to previous observations made after MI in adult animals^6,25^. These data suggest that mammalian neonatal heart regeneration yields cardiac muscle is that biomechanically integrated at the tissue level with the native ventricle after ischemic injury, a result that may have important implications on the long-term durability of the regenerated tissue.

The biomechanical properties of the LV during post-infarct remodeling are known to play an essential role in shaping outcomes after MI in adult animals^9^. A fine biomechanical equilibrium must be achieved, as infarcted myocardium that is overly compliant will bulge outward with each contraction, resulting in poor systolic function, while infarcted myocardium that is too stiff may result in impaired diastolic filling. As such, various therapeutic strategies have been developed to optimize LV biomechanics after MI. Some attempt to directly alter LV biomechanical properties, such as injectable biomaterials to stiffen the infarcted tissue^26^, or implantable patches that change the infarcted tissue’s anisotropy^27^. Others attempt to stimulate myocardial repair to reduce infarct size and collagen content^28,29^, as these two factors are the most important determinants of LV biomechanics after MI^25,30^. None of these strategies fully eliminate the infarct scar, however, and thus the difficult balance between LV compliance and stiffness remains a potential pitfall. Indeed, our team has previously investigated the impact of various exogenous myocardial repair-based therapies on LV biomechanics after MI, including tissue-engineered constructs^31,32^, and angiogenic cytokines^33–36^, observing variable degrees of biomechanical recovery but often not a full normalization of mechanical properties.

Consistent with previous studies of natural heart regeneration using a neonatal mouse MI model^15–17^, we observed that P1 mice develop minimal collagen scar by 4 weeks following MI, while P7 mice exhibit significant replacement of cardiac muscle with collagen scar. These findings were supported by echocardiographic results that illustrated a geometrically and functionally normal LV following P1 MI, but profound contractile dysfunction and adverse ventricular remodeling following P7 MI. Because neonatal heart regeneration appears to potentially yield complete restoration of the LV musculature after ischemic injury, confirmation is needed that the regenerated LV is biomechanically normal despite the initial ischemic insult, and that preservation of native LV geometry and function is ultimately associated with preservation of native LV biomechanics.

With over 20 animals per experimental group, our study was strongly powered to detect a potential difference in the stiffness between sham and post-MI neonatal mouse LV tissue. Indeed, we observed an immediate separation of the average stress-strain curves for the P7 sham and P7 MI hearts, with modulus values of 429.2 kPa and 691.6 kPa, respectively. However, no difference was detected among the average stress-strain curves for the P1 sham and P1 MI hearts through the physiologic range of wall stresses up to 10 kPa^37^, with modulus values of 363.4 kPa and 341.9 kPa, respectively. Thus, our study corroborates the results of a prior biomechanical analysis of regenerated heart muscle after cryoinjury in adult zebrafish^24^. The advantage of our study, however, involves the use of a more clinically-relevant LAD ligation technique and a more translationally-relevant mammalian animal model. Indeed, LAD ligation induces myocardial ischemia and better simulates the pathophysiology of MI and ischemic cardiomyopathy than cryoinjury, which may in fact produce an injury response distinct from that of ischemia^38,39^. Furthermore, the 4-chambered mouse heart is structurally and physiologically similar to the human heart, while the 2-chambered zebrafish heart represents a more evolutionarily distant model^40^.

In addition, our study represents, to our knowledge, the first application of lenticular hydrostatic deformation testing to the evaluation of cardiac biomechanics. Lenticular hydrostatic deformation testing, also called bulge testing, bubble testing, or membrane inflation testing in the literature, has previously been utilized to study the biomechanical properties of various biologic tissues such as skin, artery wall, pericardium, and mesentery^41–43^. We applied this technique to the heart by isolating and unfurling the LV free wall of young mice, loading the ventricle through the full range of physiologic murine blood pressures (up to 150 mmHg)^44^, and modeling the inflated LV as the cap section of a sphere. Under these conditions, we were able to generate tissue stresses well beyond the physiologic peak wall stress of adult mouse hearts after MI (<10 kPa)^37^. It is important to note that, because our system tests the passive biomechanical properties of the LV tissue, it was unnecessary to replicate the 20% strains observed *in vivo* in the contracting heart to generate physiologic wall stress levels^45^. Because the small size of the neonatal mouse heart makes planar biaxial tensile testing difficult due to the risk of generating non-homogeneous stress states during tissue stretching^46^, lenticular hydrostatic deformation testing served as a useful method for measuring the multiaxial stiffness of these tissue samples in which the region of biomechanical interest is small.

Nevertheless, our study is limited by some constraints associated with lenticular hydrostatic deformation testing and the small size of the neonatal mouse heart. Although we pressurized and loaded the LV samples during biomechanical testing, the stresses and strains experienced by the tissue were due to LV expansion and not LV contraction. This limitation, however, is shared by all *ex vivo* biomechanical testing strategies, including uniaxial and biaxial tensile testing, and micropipette aspiration. *In vivo* analyses using cardiac magnetic resonance imaging or speckle-tracking echocardiography may allow for biomechanical studies under physiologic conditions^47^, but the small size of the neonatal mouse heart introduces significant issues with imaging resolution. Due to similar heart size limitations and the risk of leaking during pressurization, we were also unable to perform time-course biomechanical analyses using lenticular hydrostatic deformation testing starting at an earlier age. In addition, lenticular hydrostatic deformation testing typically assumes a thin, membrane-like sample in which the ratio of tissue thickness to the radius of curvature during inflation is 0.1 or less. While LVWTd of our heart samples ranged from 0.24 mm to 0.92 mm, the model sphere radius during inflation was typically 2.5–3.0 mm, and thus the neonatal mouse LV did not always behave as an ideal thin membrane. The consequence, however, is that we would expect the modulus to be overestimated for thicker samples^48^, for which the P1 sham and P1 MI hearts would be equally affected. Moreover, using the Lamé equation^48^, we predict this minor source of error to be less than 10%, and we nevertheless found that the thinner P7 MI hearts exhibited significantly greater tissue modulus values than the thicker P7 sham hearts, indicating that our ability to detect a difference in modulus is not compromised. Our testing system also included only one camera’s point-of-view, and we therefore had to assume that the LV samples were biomechanically isotropic^49^. Because myocardial tissue is anisotropic in nature^50^, we expect some degree of error in the absolute values of our modulus data, but again, the comparative similarities or differences in modulus between the experimental groups are unlikely to be significantly affected, especially given the strong statistical power of our study. Finally, we were not able to confirm successful P1 MI using echocardiography during the first days after surgery due to inadequate imaging resolution and high rates of maternal cannibalism after attempting early echocardiography^51^. However, our team possesses extensive experience with neonatal rodent surgery^17,19,52^, and we designed our study to include several other experiments to confirm MI and subsequent regeneration (including fluorescent lectin perfusion, Masson’s trichrome and EdU analysis, and late echocardiography). Finally, we also included a large sample size for our biomechanical analysis to improve the strength of our data.

Overall, natural heart regeneration after MI in neonatal mice results in preserved native LV biomechanical properties at 4 weeks after injury. Our result suggests that naturally regenerated cardiac muscle may be biomechanically integrated at the tissue level with the native LV, thereby strengthening its translational potential as a future therapy for patients suffering MI or ischemic cardiomyopathy.

## Methods

### Animal care and biosafety

Pregnant female C57BL/6J mice were obtained from Charles River Laboratories (Wilmington, MA, USA) and monitored for parturition at minimum every 12 hours. Neonatal pups were kept with their nursing mother until age 21 days old, at which time the pups were weaned. Food and water were otherwise provided ad libitum. All experiments involving animals were performed in accordance with the United States National Institutes of Health “Guide for the Care and Use of Laboratory Animals” (8th Edition, 2011). All procedures involving animals were approved by the Institutional Animal Care and Use Committee at Stanford University (Protocol 28921).

### Neonatal mouse myocardial infarction model

At age P1, defined as 12–24 hours after parturition was observed, neonatal mice (n = 63) underwent permanent LAD coronary artery ligation or sham surgery. Both male and female pups were included in this experiment. The P1 pups were removed from their mother’s nest immediately prior to surgery. Each pup was wrapped in gauze and placed in an ice bath for 6 minutes to induce hypothermic circulatory arrest^17,51,52^. The anesthetized pup was then placed supine on the operating field. The chest was prepped with betadine solution and ethanol, and a fourth-interspace left anterior thoracotomy was performed using microscope guidance to expose the heart. A 6–0 polypropylene suture was used to ligate the LAD artery 1 mm below the left atrial appendage. Sham controls underwent the same procedure, including hypothermic circulatory arrest and passage of the needle through the myocardium below the LAD, but without tying the suture. Chest closure was performed in layers using interrupted 6–0 polypropylene sutures. A drop of 2% lidocaine was placed within the incision just prior to skin closure. No other analgesics were required^51,53^. The pups were recovered on a 37 °C warm pad. Once all pups in the litter were awake and active after surgery, they were returned to their mother’s care.

At age P7, defined as between 7–8 days after parturition was observed, neonatal mice (n = 46) underwent LAD ligation or sham surgery under hypothermic circulatory arrest using the same technique as described above, except that the time on ice was 7 minutes for each pup.

For both age groups, the mice were randomly assigned to receive LAD ligation or sham surgery. Prior to the start of surgery, the purpose of each litter was assigned as either (1) confirmation of LAD ligation, (2) histological analyses, or (3) biomechanical testing. A flowchart illustrating the use of animals over the course of the study is shown in Supplemental Fig. 2.

### Injection of fluorescent-labeled lectin

One hour after fully recovering from LAD ligation or sham surgery, a subset of P1 mice were sacrificed to confirm successful MI. The neonate mice were deeply anesthetized using 4% inhaled isoflurane (Fluriso, Vet One, Boise, ID, USA) and euthanized by decapitation. The heart was exposed via median sternotomy, and 50 μL of fluorescent-labeled lectin (0.25 mg/mL, DyLight 649-labeled *Lycopersicon esculentum* tomato lectin, Vector Laboratories, Burlingame, CA, USA) was injected into the right ventricle. The heart was explanted and incubated in the dark for 45 minutes in 4% paraformaldehyde (PFA) at 4 °C. The hearts were then washed 3 times with phosphate-buffered saline (PBS) and placed in Vectashield (Vector Laboratories, Cat: H-1000) for 1–2 hours at room temperature before storing at −20 °C until use.

### Whole-mount confocal microscopy of lectin-perfused neonatal hearts

Whole P1 mouse hearts were placed with the anterior wall facing up on a double-concave microscope slide (Sail brand, Cat: 7104). The heart was then flattened with a microscope coverslip (Fisherbrand, Cat: 12545 F, Pittsburgh, PA, USA). Images were captured using an inverted Zeiss LSM 780 multiphoton laser scanning confocal microscope (10X and 20X objectives, Zeiss, Oberkochen, Germany). Image processing and analysis was performed manually using Zeiss Zen software.

### Injection of cell proliferation marker

On postoperative days 1, 7, and 14, a subset of P1 mice assigned for histological analyses received EdU (Thermo Fisher Scientific, Cat: A10044, Waltham, MA, USA) to assess cell proliferation^54^. The mice were anesthetized using 2–3% inhaled isoflurane. EdU (20 mg/kg dose, 2.5 mg/mL final concentration in 10% dimethyl sulfoxide in PBS) was injected subcutaneously beneath the dorsal skin. The mice were recovered on a 37 °C warm pad for 30 minutes, and then returned to their mother’s care once all pups in the litter were awake and active.

### Heart explant and sample preparation for histology

At 4 weeks after surgery, mice assigned for histological analyses were deeply anesthetized using 4% inhaled isoflurane and euthanized by cervical dislocation. The heart was exposed via median sternotomy and arrested using potassium chloride (1 mEq/kg) injected directly into the right ventricle. The explanted hearts were flushed with PBS, filled with optimum cutting temperature compound (OCT, Fisher HealthCare, Cat: 23730571, Houston, TX, USA), and finally embedded in OCT using 2-methyl butane on dry ice. The samples were stored at −80 °C until use.

### Masson’s trichome staining

For Masson’s trichrome staining (American MasterTech, Cat: KTMTR2PT, Lodi, CA, USA), one LV short-axis section (10 μm thickness) at the level of the mid-lower papillary muscles was selected for each heart. Briefly, the samples were thawed at 4 °C, rehydrated with ethanol and fixed in Bouin’s solution (Polysciences Inc., Cat: 16045-1, Warrington, PA, USA) at 60 °C, and then washed with tap water until the tissue cleared. Tissue sections were incubated in hematoxylin solution, followed by Scarlet-Acid Fuchsin, then rinsed and incubated in phosphotungstic/phosphomolybdic acid solution. Next, the sections were stained with Aniline Blue at room temperature, incubated in 1% acetic acid (not provided by the kit), and finally washed with tap water. The sections were dehydrated and mounted in Cytoseal (Thermo Fisher Scientific, Cat: 8310-16), and the finished slides were imaged using an EVOS XL Core Imaging System (Thermo Fisher Scientific, Cat: AMEX-1000). Infarct scar size was quantified using ImageJ software (National Institutes of Health) by measuring the area of fibrosis stained in blue as a percentage of total LV area.

### EdU and Immunohistochemistry

For assessment of EdU uptake by troponin^+^ cardiomyocytes, one LV short-axis section (10 μm thickness) at the level of the mid-lower papillary muscles was chosen for each heart. The sections were incubated with 4% PFA at room temperature, rinsed with 3% bovine serum albumin in PBS, and then incubated in 0.5% PBS-Tween. The sections were rewashed, and then incubated with 0.5 mL of the Click-iT reaction cocktail according to the kit protocol (Click-iT EdU Cell Proliferation Kit for Imaging, Thermo Fisher Scientific, Cat: C10339). For troponin immunohistochemistry, the sections were incubated with anti-cardiac troponin I primary antibody (1 mL, 1:200, Abcam, Cat: ab47003, Cambridge, MA, USA) at 37 °C for 90 minutes, washed with PBS, and then incubated with goat anti-rabbit IgG H&L Alexa Fluor 488 secondary antibody (1 mL, 1:200, Abcam, Cat: 150077) at 37 °C for 90 minutes in the dark. Finally, the sections were incubated with DAPI (NucBlue Fixed Cell ReadyProbes Reagent, ThermoFisher Scientific, Cat: R37606). The finished slides were kept at 4 °C until ready for imaging.

The heart sections were imaged using an inverted Zeiss LSM 780 multiphoton laser scanning confocal microscope (10X and 20X objectives). Captured Z-stacked images were processed and manually analyzed using Zeiss Zen software. Cells positive for troponin, EdU, and DAPI were quantified over 3 randomly selected regions (125 μm × 90 μm) of each 20X field of view (425 μm × 425 μm). The cell counts were averaged and finally extrapolated to determine the total EdU^+^/troponin^+^ cells per 20X region of interest.

### Echocardiography

Transthoracic echocardiography was performed 4 weeks after surgery for all mice assigned for biomechanical testing. After anesthetizing the animals using 2% inhaled isoflurane, a Vevo 2100 imaging system equipped with an ultra-high frequency linear array transducer (MicroScan MS250 13–24 MHz transducer, VisualSonics Inc., Toronto, Canada) was used to acquire left parasternal LV short- and long-axis images of the heart. Measurements of LV geometry and function over the infarct zone at the level of the mid-lower papillary muscles were performed using Vevo Lab software (VisualSonics Inc.). Echocardiographic metrics included LV wall thickness in diastole, LV internal diameter in systole and diastole, and LV ejection fraction. Image acquisition and analysis were each performed by a single individual to limit interobserver bias.

### Heart explant and sample preparation for biomechanical testing

After echocardiography at 4 weeks post-surgery, the mice assigned for biomechanical testing were deeply anesthetized using 4% inhaled isoflurane and euthanized by cervical dislocation. The heart was exposed via median sternotomy and arrested in diastole using University of Wisconsin (UW) cold cardioplegic storage solution (Bridge to Life Ltd., Columbia, SC, USA), injected directly into the right ventricle. The heart was explanted, and the right ventricle and interventricular septum were excised, leaving the unfurled LV free wall intact. The samples were stored in UW solution at 4 °C until use to avoid contracture.

### Lenticular hydrostatic deformation testing

Biomechanical testing of the LV tissue was performed using a custom-built, plexiglass apparatus encasing a pressurizable chamber with an inflow cannula and high-fidelity pressure transducer (Model 210, Precision Measurement Co., Ann Arbor, MI, USA) to simulate pressure loading on the myocardium^41,55^. A motor-controlled syringe plunger (1705 RN, Hamilton Co., Reno, NV, USA) was used to deliver 37 °C PBS through the inflow cannula into the chamber to deair the system. LV tissue samples were then mounted flat atop the chamber, such that the anterolateral LV wall distal to the LAD ligation stitch (i.e. the infarct zone) was positioned directly over the 5 mm orifice on the roof of the chamber. The system was then sealed with a gasket placed directly over the LV sample and locked down with screws to prevent leakage (Fig. 4a). Using 37 °C PBS delivered at a rate of 0.5 μL/s, the system was pressurized to 150 mmHg, encompassing the full physiologic blood pressure range for healthy adult C57BL/6J mice^44^. Lenticular deformation of the LV wall due to increasing hydrostatic pressure within the chamber was tracked in profile (Fig. 4b,c), using a high-resolution camera (Chameleon3 USB3, model CM3-U3-50S5M-CS, FLIR Systems, Wilsonville, OR, USA). All samples were tested using the same biomechanical testing protocol within 2 hours of heart explant. A schematic illustration of lenticular hydrostatic deformation testing is shown in Fig. 4d.

### Biomechanical modeling and data analysis

The pressurized LV wall was modeled as a thin-walled spherical cap with uniform multiaxial stress and curvature. Thus, the stress on the LV wall (*σ*) for each sample was given by the equation below, where *p* is the pressure load measured by the pressure transducer, *R* is the radius of the model sphere, and *t* is the tissue thickness under stress.

1 $$\sigma =\frac{pR}{2t}$$

Using the Pythagorean theorem, the radius of the model sphere (*R*) was defined by the following formula, where *h* is the maximum height of lenticular deformation, and *a* is the radius of the chamber orifice over which the sample is mounted (*a* = 2.5 mm).

2 $$R=\frac{{h}^{2}+{a}^{2}}{2h}$$

The tissue thickness under stress (*t*) was defined by the formula below, assuming constant volume deformation, where *t*_0_ is the tissue thickness in diastole measured by echocardiography for each individual sample prior to mechanical testing, and *λ* is the deformation stretch.

3 $$t=\frac{{t}_{0}}{{\lambda }^{2}}$$

The deformation stretch (*λ*) was further determined by the following equation, where *L* is the arc length of deformation.

4 $$\lambda =\frac{L}{2a}$$

The true multiaxial strain (*ε*) for each sample was calculated as:

5 $$\varepsilon =\,\mathrm{ln}(\lambda )$$

Finally, the composite multiaxial modulus (*E*) was calculated using the formula below, where *B* is the slope of the linear aspect of the stress-strain curve, and *v* is Poisson’s ratio, which was approximated as *v* = 0.5 for nearly incompressible materials such as cardiac and other biologic tissues^33,56,57^.

6 $$E=B(1-v)$$

### Statistical analysis

All research team members remained blinded to the experimental group assignments after surgery until data collection was completed. Statistical analyses were performed using Stata version 14.2 (StataCorp LLC., College Station, TX, USA). Continuous variables were reported as mean ± standard error and compared using 2-sample t-tests. A p-value < 0.05 was considered statistically significant. Observations for neonate males and females did not differ.

## Supplementary information

Supplementary information.

## Data Availability

Data will be made available upon reasonable request.
