# Supplementary material for: Multiaxial Lenticular Stress-Strain Relationship of Native Myocardium is Preserved by Infarct-Induced Natural Heart Regeneration in Neonatal Mice
Source: Sci Rep. 2020 Apr 30;10:7319. doi: 10.1038/s41598-020-63324-w (PMC7193551; doi:10.1038/s41598-020-63324-w)
Supplement: Supplementary file 1 — Supplementary information. [file 41598_2020_63324_MOESM1_ESM.pdf]

Multiaxial Lenticular Stress-Strain Relationship of Native Myocardium is Preserved by Infarct-Induced Natural Heart Regeneration in Neonatal Mice

Hanjay Wang[1,2], Ross Bennett-Kennett[3], Michael J. Paulsen[1], Camille E. Hironaka[1], Akshara D. Thakore[1], Justin M. Farry[1], Anahita Eskandari[1], Haley J. Lucian[1], Hye Sook Shin[1], Matthew A. Wu[1], Annabel M. Imbrie-Moore[1,4], Amanda N. Steele[1,5], Lyndsay M. Stapleton[1,5], Yuanjia Zhu[1,5], Reinhold H. Dauskardt[3], Y. Joseph Woo[1,2,5*]

[1] Department of Cardiothoracic Surgery, Stanford University, Stanford CA

[2] Stanford Cardiovascular Institute, Stanford University, Stanford CA

[3] Department of Materials Science and Engineering, Stanford University, Stanford CA

[4] Department of Mechanical Engineering, Stanford University, Stanford CA

[5] Department of Bioengineering, Stanford University, Stanford CA

***Corresponding Author:**

Y. Joseph Woo, MD

Department of Cardiothoracic Surgery, Stanford University

Falk Building CV-235, 300 Pasteur Drive, Stanford, CA 94305-5407, USA

Phone: (650) 725-3828; Fax: (650) 725-3846; E-Mail: joswoo@stanford.edu

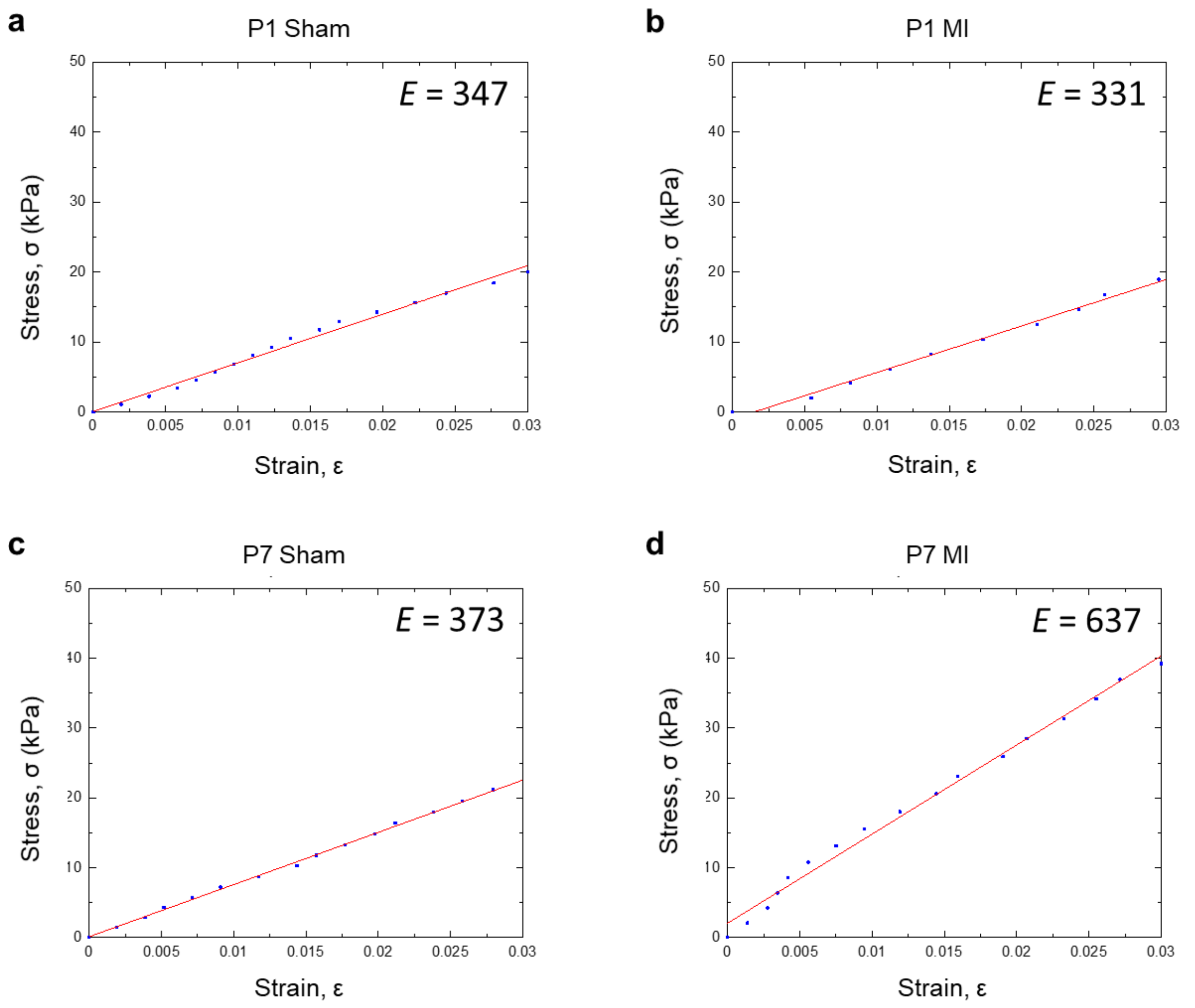

Supplemental Figure 1. Experimental Stress-Strain Curves. Representative stress-strain curves for left ventricular tissue after sham or myocardial infarction (MI) surgery in neonatal mice on postnatal day 1 (P1) or postnatal day 7 (P7), derived from lenticular hydrostatic deformation testing at 4 weeks after surgery. One representative heart from the **(a)** P1 sham group, **(b)** P1 MI group, **(c)** P7 sham group, and **(d)** P7 MI group were selected. The composite multiaxial modulus (E) is indicated for each sample.

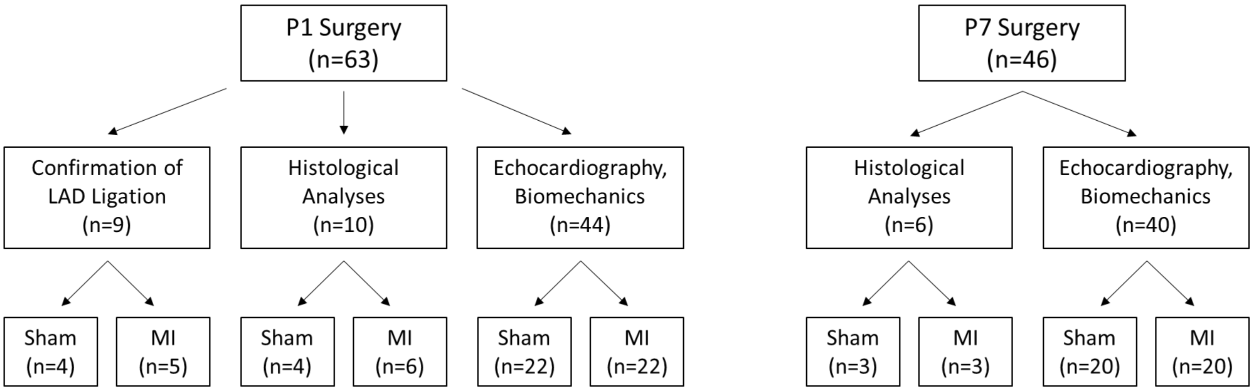

Supplemental Figure 2. Study Flowchart. Sham or myocardial infarction (MI) surgery was performed in neonatal mice either on postnatal day 1 (P1) or postnatal day (P7). Mice were designated to undergo either assessment of successful left anterior descending (LAD) coronary artery ligation, or histological analyses, or echocardiography and lenticular hydrostatic deformation testing.
